# Long-term/home parenteral nutrition: expert consensus statements regarding intravenous lipid emulsions

**DOI:** 10.3389/fnut.2025.1718116

**Published:** 2025-12-08

**Authors:** Manpreet S. Mundi, Robert G. Martindale, Philip C. Calder, Stanislaw Klek

**Affiliations:** 1 Division of Endocrinology, Diabetes, Metabolism and Nutrition, Mayo Clinic, Rochester, MN, United States; 2 Department of Surgery, Oregon Health and Science University, Portland, OR, United States; 3 Faculty of Medicine, University of Southampton, Southampton, United Kingdom; 4 NIHR Southampton Biomedical Research Centre, University Hospital Southampton NHS Foundation Trust and University of Southampton, Southampton, United Kingdom; 5 Surgical Oncology Clinic, The Maria Sklodowska-Curie National Cancer Institute, Krakow, Poland

**Keywords:** consensus, fish oil, guidelines, home parenteral nutrition, intestinal failure, lipids, omega-3 fatty acids, palliative nutrition

## Abstract

Home parenteral nutrition (HPN) is the primary life-saving therapy for patients with chronic intestinal failure (CIF). Patients requiring palliative nutrition, such as those with advanced cancer, may also benefit from HPN. Lipids are an integral part of parenteral nutrition (PN), but the use of intravenous lipid emulsions (ILEs) in PN continues to raise numerous questions for clinicians despite improved understanding and knowledge. The Lipids in PN Summit involved a panel of international experts with extensive research and clinical experience in use of PN. They assessed the current state of knowledge and developed expert consensus statements regarding the use of ILEs in patients requiring PN. The statements are also provided to help bridge the gaps between evidence and clinical practice, hence complementing formal societal guideline recommendations for the use of PN. This review briefly summarizes the rationale for considering ILE choice as a central component of any strategy for HPN patients, and discusses aspects of special interest within the context of HPN and long-term PN use in general such as essential fatty acid (EFA) delivery, the prevention of IF-associated liver disease (IFALD), and clinical evidence within HPN populations. In particular, potential clinical advantages of modern composite ILEs containing fish oil are reviewed, with biological effects of omega-3 polyunsaturated fatty acids (PUFAs) imparting additional clinical benefits. A future perspective section shares some proposals to address the difficulties of data generation within HPN, and suggested approaches to take as part of current clinical practice in the absence of definitive data. For now, the existing body of evidence should provide the basis for clinical care, and where evidence is lacking expert recommendations must suffice. The consensus statements from the Lipid Summit aim to summarize aspects mostly relevant for everyday clinical care; those relevant to the HPN setting are presented in this review.

## Introduction

Intestinal failure (IF) can be defined as a “reduction of gut function below the minimum necessary for the absorption of macronutrients and/or water and electrolytes, such that intravenous supplementation is required to maintain health and/or growth” (1, 2). Chronic IF (CIF) occurs when IF persists for months or years (2). CIF can be caused by either severe gastrointestinal or systemic benign diseases, or end-stage intra-abdominal or pelvic cancers, and typically requires long-term parenteral nutrition (PN) (2). In Europe, CIF caused by benign disease has been classified as a rare disease (3), with estimated prevalence rates for CIF patients receiving PN of 5–80 cases per million (1, 4, 5). Home parenteral nutrition (HPN) is the primary life-saving therapy for patients with CIF (6). HPN is typically limited to metabolically stable patients with CIF who are capable, willing, and trained for managing PN outside an acute hospital setting (7). In addition, HPN may be given as palliative nutrition in some patients such as those with cancer, or is sometimes used to prevent or treat malnutrition in patients with a functioning intestine who decline medical nutrition via the oral/enteral route (2, 6, 8). While some patients may use PN as their sole source of nutrition, others may receive a combination of PN and oral/enteral nutrition (9).

A fairly complete population-based evaluation from France with data derived from the National Health Insurance database in 2019 reported for adults an incidence of HPN of 220 cases per million and an overall prevalence of 253 per million inhabitants (10). In general, however, the number of patients receiving HPN often remains difficult to ascertain, as evaluations are frequently affected by methodological shortcomings (10). Moreover, considerable variations in HPN use occur between countries, particularly in situations such as HPN use in cancer patients (2). In some countries, a continuous increase in HPN has occurred over recent years [e.g., the United Kingdom (UK) and Poland] (10, 11), while concurrently, HPN use has dropped in other countries [e.g., the United States of America (USA)] (5).

Lipids are an integral part of PN in all settings where PN is required (12), but the use of intravenous lipid emulsions (ILEs) in PN continues to raise numerous questions for clinicians despite improved understanding and knowledge. These include: essential fatty acid (EFA) delivery, the prevention of IF-associated liver disease (IFALD), and a relative lack of clinical evidence within HPN populations, with all of these being of special interest within the context of HPN and long-term PN use in general. These aspects are addressed as part of this review, which is derived from the international Lipids in Parenteral Nutrition Summit, held on November 3 and 4, 2022, in New Orleans, USA, involving a panel of international experts with clinical and scientific experience of PN to discuss biological and clinical aspects of lipids used in PN (12). Consensus statements were produced with the aim of providing practical guidance regarding the use of lipid emulsions in PN to complement societal nutrition guidelines (2, 6, 13). A full set of these consensus statements has been published previously (12). Table 1 shows a subset of these consensus statements relevant to this review/the HPN setting.

**Table 1:** Consensus statements for intravenous lipid emulsion (ILE) use in adults receiving parenteral nutrition (PN) relevant to patients with chronic intestinal failure or other patients requiring long-term parenteral nutrition^a^.

Consensus statement	Voting
Chronic intestinal failure and long-term parenteral nutrition	
In patients requiring long-term parenteral nutrition, ILEs are an integral part of parenteral nutrition.	Agree: 18 (100%) Do not agree: 0 Do not wish to answer: 0
There is accumulating scientific evidence from clinical trials to indicate that ILEs containing fish oil are preferred over ILEs derived exclusively from soybean oil for adult home parenteral nutrition patients or other long-term parenteral nutrition patients.	Agree: 18 (100%) Do not agree: 0 Do not wish to answer: 0
In patients on long-term parenteral nutrition, soybean oil ILE doses should not exceed 1 g/kg/day to prevent liver complications. The risk of liver complications in adult long-term parenteral nutrition patients may be reduced by using ILEs containing fish oil.	Agree: 18 (100%) Do not agree: 0 Do not wish to answer: 0
In patients with chronic intestinal failure, when more than 1 g lipids/kg/day is required, alternative ILEs (fish oil, olive oil, MCTs) should be used to limit the amount of soybean oil provided.	Agree: 18 (100%) Do not agree: 0 Do not wish to answer: 0
A minimum dose of ILEs should be given to prevent EFA deficiency.	Agree: 18 (100%) Do not agree: 0 Do not wish to answer: 0
ILEs containing fish oil may be beneficial in patients with IFALD.	Agree: 18 (100%) Do not agree: 0 Do not wish to answer: 0
The use of mixed lipid emulsions containing soybean oil, olive oil, MCTs and/or fish oil at the recommended dose has not been shown to lead to EFAD in clinical practice. A 100% fish oil ILE has also not been shown to lead to EFAD in clinical practice.	Agree: 18 (100%) Do not agree: 0 Do not wish to answer: 0
Supplemental parenteral nutrition	
Supplemental parenteral nutrition is a combination of oral/enteral nutrition and parenteral nutrition. It may be considered as a strategy with the intent to increase macronutrient delivery and to maintain/improve the nutritional status of patients such as critically ill (acute phase), surgical, and cancer patients if oral or enteral nutrition is insufficient. ILEs are an integral part of supplemental parenteral nutrition.	Agree: 17 (100%)^b^ Do not agree: 0 Do not wish to answer: 0
Administration of supplemental parenteral nutrition through a peripheral line can be considered over a short period of time when central line access is unavailable or as a bridge until central line access is available. ILEs are an integral part of peripheral parenteral nutrition.	Agree: 17 (94.4%) Do not agree: 1 (5.6%) Do not wish to answer: 0
ILEs in parenteral nutrition—practical handling aspects	
If using all-in-one admixtures, the maximum infusion duration is 24 h.	Agree: 18 (100%) Do not agree: 0 Do not wish to answer: 0
When ILEs are given independent of dextrose and amino acids, infusion duration of ILEs should not exceed 12 h to minimize the risk of contamination.	Agree: 18 (100%) Do not agree: 0 Do not wish to answer: 0
Request to organizations issuing guidelines and recommendation in parenteral nutrition	
Nutrition societies should issue guidelines and recommendations addressing clinical validity when performing a systematic review. Differences in inclusion/exclusion criteria and methodology can result in significant differences in outcomes and conclusions. Translation of systematic review conclusions into clinical guidelines is also affected by many factors including the intent of the convening body, geographical regulations impacting clinical options, and balance between clinical requirement and need for additional definitive evidence.	Agree: 18 (100%) Do not agree: 0 Do not wish to answer: 0

^a^These consensus statements were formulated and voted on by the expert panel (listed below) participating in the International Lipids in PN Summit 2022, and thus represent the collective opinion of the summit experts informed by scientific evidence. The consensus summit experts who attended this meeting were: Magnus Bäck, Sweden; Philip Calder, UK; Sarah Cogle, USA; Valerio Chiurchiù, Italy; David Evans, USA; Leah Gramlich, Canada; Martin Hersberger, Switzerland; Stanislaw Klek, Poland; Robert Martindale, USA; Stephen McClave, USA; Bettina Mittendorfer, USA; Manpreet Mundi, USA; Maurizio Muscaritoli, Italy; Reid Nishikawa, USA; Jayshil Patel, USA; Lorenzo Pradelli, Italy; Martin Rosenthal, USA; Charles Serhan, USA; Christian Stoppe, Germany; Kelly Tappenden, USA; Dan Waitzberg, Brazil; Malissa Warren, USA; Paul Wischmeyer, USA. (Note not all experts were present for all sessions of the meeting/voted on all of the consensus statements).

^b^One delegate did not answer these questions.

EFA, essential fatty acid; EFAD, essential fatty acid deficiency; ILE, intravenous lipid emulsion; MCT, medium chain triglyceride.

## Lipid emulsions in HPN formulations

HPN should provide a comprehensive mix of nutrients, including glucose/dextrose, lipids, amino acids, vitamins, and minerals (14, 15). The primary reasons for the inclusion of lipids as part of HPN is that they provide a source of non-protein energy and supply the body with EFAs (14). As fatty acids are energy-dense nutrients, their inclusion means that less glucose/dextrose is needed as part of HPN to fulfil each patient’s energy needs (2, 15). This is advantageous as high-glucose/dextrose PN can lead to complications such as hyperglycemia and hepatic steatosis (2, 15).

Soybean oil, the primary traditional lipid source for ILEs used in PN, contains a relatively high proportion of omega-6 polyunsaturated fatty acids (PUFAs), with linoleic acid accounting for more than 50% of all fatty acids supplied (12, 16). Lipid mediators derived from linoleic acid and its metabolite arachidonic acid (e.g., eicosanoids) are involved in inflammatory pathways and may suppress the cell-mediated immune response (12, 16, 17). More recent generations of ILEs contain a mixture of different lipids to reduce omega-6 PUFA content and thus lower the likelihood of contributing to inflammatory and/or immunosuppressive processes (12, 16). These composite ILEs typically combine soybean oil with one or more alternative lipids, such as medium-chain triglycerides (MCTs), olive oil, and fish oil (16).

The optimal quantity of lipids to give as part of HPN has not been established with precision (14). It is widely accepted, however, that in the case of long-term HPN treatment (more than six months), the amount of intravenous soybean oil lipid emulsion (regardless of ILE source) should not exceed 1 g/kg per day (2, 12, 14). Thus, alternative ILEs (blends with fish oil, olive oil and/or MCTs) are recommended to limit the amount of soybean oil provided and reduce the risk of IFALD (Table 1, statements 27 and 28), as well as providing other potential clinical benefits, as detailed later in this review (2, 12).

## EFA coverage

A minimum dose of ILEs should be given to prevent EFA deficiency (Table 1, statement 29) (12). According to the American Society for Parenteral and Enteral Nutrition (ASPEN), EFA deficiency (EFAD) can be prevented by providing 2–4% of energy from linoleic acid and 0.25–0.5% of energy from *α*-linolenic acid (13). The recommendation from the European Society for Clinical Nutrition and Metabolism (ESPEN) is more practical: a minimum quantity of 1 g/kg/week ILE should be given to prevent EFAD during long-term PN (2). This quantity was originally established for soybean oil, but no differences were found in EFA status in studies conducted with the same quantity of lipids derived from other lipid sources and/or composite ILEs (2, 18–20).

**Figure 1:**
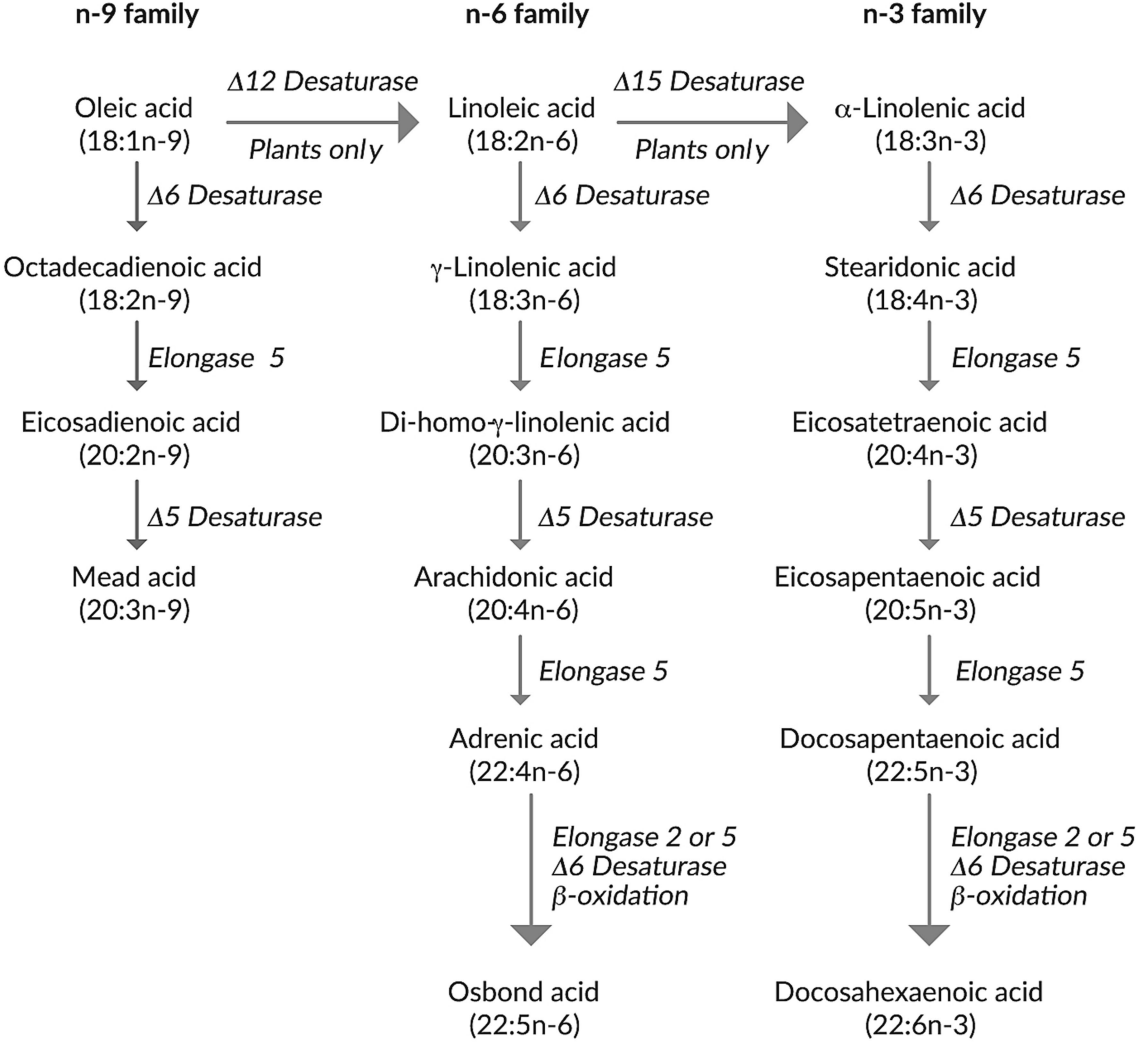
Metabolic processing of omega-3 (n-3), omega-6 (n-6), and omega-9 (n-9) polyunsaturated fatty acids.

The EFA concept, which derives from the 1920s, has been revisited recently alongside the availability of composite ILEs (21, 22). Traditionally, two fatty acids (the omega-6 PUFA linoleic acid and the omega-3 PUFA *α*-linolenic acid) were considered as essential (i.e., must be supplied exogenously) because the body cannot produce them on its own and these are obtained from dietary plant sources (12, 16, 23). However, we now know that α-linolenic acid may also be substituted with its downstream products eicosapentaenoic acid (EPA) and docosahexaenoic acid (DHA), which are available in fish oil (12, 21, 22, 24). Notably, some reports have shown that rodents fed a diet in which the only PUFAs provided were DHA and arachidonic acid (ARA) did not develop biochemical or clinical EFAD over many generations, at least in part because of retroconversion pathways (22). These include retroconversion of ARA to linoleic acid and retroconversion of DHA into its upstream metabolites, EPA and docosapentaenoic acid (DPA) (22) (Figure 1).

In clinical practice, neither the use of ILEs containing soybean oil, olive oil, MCTs, and/or fish oil, given at the recommended dosages, nor the use of 100% fish oil ILE in adults (e.g., because of a soybean oil allergy) have resulted in symptoms of EFAD—either with short- or long-term use (Table 1, statement 31) (20, 22, 25, 26). In adults, EFAD cases in association with PN have mainly been reported during the era of ILE-free PN in the USA (27, 28). EFAD is rare in adults, and generally only occurs if lipid intake, digestion, absorption, and/or metabolism, are impaired significantly (12, 29). EFA monitoring is advised in these particular situations. The most common marker for EFA status is the ratio of mead acid (triene) to arachidonic acid (tetraene), though thresholds indicating EFAD are inconsistent (with >0.2 being proposed for biochemical EFAD and >0.4 proposed for clinical symptoms) (22, 26, 30). Typically, the same desaturase and elongase enzymes metabolize the omega-3, omega-6, and omega-9 PUFAs (Figure 1). If there is a deficiency in omega-3 and omega-6 PUFAs, then oleic acid is metabolized to mead acid, increasing the ratio of mead acid to arachidonic acid. With the arrival of newer ILEs, the usefulness of this ratio has been criticized, as it does not take into account varying ILE compositions (22, 26). As an example, patients given an ILE containing olive oil and soybean oil may have a rise in mead acid owing to the metabolism of the (relatively high) oleic acid content of olive oil in the ILE (19) (Figure 1). Thus, a good understanding of ILE fatty-acid composition and metabolic pathways (Figure 1) is necessary if EFA status is monitored based on the triene-to-tetraene ratio. In addition to biochemical findings on laboratory tests, clinical signs of EFAD may include dermatitis (scaling, thinning, and dry skin), alopecia, and neurological or hematological effects, and in extreme/rare cases may even lead to death (2, 31–34). However, it should be noted that these symptom descriptions stemmed from pediatric patients (31), adults with severe fat malabsorption (32), following the use of lipid-free PN (33), or findings were inconclusive and thus extrapolated from rodents (34).

## Prevention of IFALD

The development of IFALD – a serious and potentially life-threatening complication – remains a concern with long-term PN (12). The term IFALD has replaced older terminologies such as PN-associated liver disease (PNALD) and PN-associated cholestasis (PNAC) (35). Whilst there is still no generally agreed definition of IFALD, ESPEN have proposed that “the term IFALD refers to liver injury as a result of one or more factors relating to IF including, but not limited to, PN and occurring in the absence of another primary parenchymal liver pathology (e.g., viral or autoimmune hepatitis), other hepatotoxic factors (e.g., alcohol/medication) or biliary obstruction” (36, 37).

IFALD is associated with a variable presentation, and can include hepatic steatosis, cholestasis, cholelithiasis, and hepatic fibrosis (38). In some cases, IFALD may resolve despite continuing HPN, though this has been observed more for steatosis and cholestasis and less with fibrosis (typically a feature of later disease stages) (39). A clinical diagnosis of IFALD is often made because of the presence of abnormal liver function tests, evidence of radiological abnormality, or (rarely) because of histological abnormalities in the absence of other primary pathology (37, 38). Currently, there are neither agreed criteria for the diagnosis of IFALD, nor established means for assessing disease severity, progression, and/or response to treatment (40).

IFALD pathogenesis is complex, with IF-, PN-, and systemic-related factors all playing a role (36). Risk factors for IFALD development and progression include sepsis, inflammation, excessive energy intake, long duration of PN, and long-term use of soybean-oil ILEs as part of PN (28, 36, 37). Pure soybean-oil ILEs are considered a risk factor when given at doses above 1 g/kg/day over prolonged periods (i.e., for >6 months), resulting in less frequent use of ILEs (41, 42). Hence, when higher doses of ILEs are required, composite ILEs (consisting of a blend of lipids instead of pure soybean oil) should be considered (Table 1, statements 27 and 28) as mentioned earlier (2, 12). This, and other proposed IFALD risk minimization measures, are summarized in Table 2 (2, 36, 37).

**Table 2:** Proposed measures to prevent the development and progression of intestinal failure associated liver disease (IFALD) (2, 36, 37).

Prevention and timely management of sepsis. Avoidance of hepatotoxic agents, including medications and alcohol. Attempt to preserve intestinal length and/or to retain the colon in-continuity. Maintain oral or enteral intake. Consider cycling PN. Avoid overfeeding (excess of macronutrients), and deficiency of micronutrients. Limit the prescription of pure soybean-oil ILEs to less than 1 g/kg/day. If more ILE is required then use composite ILEs (i.e., those containing blends of lipids). Abstain from infusion rates of ILEs exceeding 0.11 g/kg/h in order to avoid fat overload syndrome

ILE, intravenous lipid emulsion; PN, parenteral nutrition.

In adults, pure soybean-oil ILEs supplied in excess of 1 g/kg/day have been associated with liver damage, and in particular IFALD cholestasis (36). Some mechanisms that have been implicated in this phenomenon are (i) the activation of Kupffer cells following the use of pure soybean oil ILEs, (ii) soybean oil contains high levels of omega-6 PUFAs, which can cause inflammation (iii) PUFA peroxidation, (iv) low *α*-tocopherol content (a major lipophilic antioxidant agent), (v) fat overloading, and (vi) high plant sterol content (especially sigmasterol), as found in soybean oil ILEs, that affect the bile metabolism pathway and lead to decreased bile flow (36). In contrast, composite ILEs contain a variety of lipids to reduce omega-6 PUFA content and thus lower the likelihood of excessive inflammation (12). In this context, the omega-3 PUFAs, EPA and DHA, that are abundant in fish oil are of particular interest owing to their inflammation-resolving properties, immunomodulatory effects, and reduced oxidative properties (12). Moreover, ILEs containing fish oil tend to have higher *α*-tocopherol content and less phytosterol content than ILEs without fish oil, which may also be beneficial (36, 43).

Modifying the risk of development and/or progression of IFALD oil by replacing pure soybean-oil ILEs with composite ILEs containing fish oil has not yet been evaluated using long-term clinical studies in adults. Nevertheless, there are some case studies showing composite ILEs containing fish oil have improved IFALD in adult patients (38, 44, 45). Improvement in IFALD was typically accompanied by favorable effects on liver function parameters and also glucose/dextrose intake could be reduced (38, 44, 45). One early case study may help to illustrate how clinical management has evolved (44). When the patient developed IFALD, provision of soybean ILE was decreased and glucose/dextrose supply was increased to provide similar levels of non-protein energy. This approach led to an improvement in IFALD, but (unsurprisingly) the patient developed signs of insulin resistance. IFALD symptoms worsened again when attempts were made to increase ILE provision in order to reduce the quantity of glucose/dextrose supplied. Finally, the patient was given a composite ILE containing fish oil, IFALD symptoms improved, and total bilirubin levels were normalized (44). A follow-up investigation switched patients who were intolerant of a soybean oil ILE (*n* = 64) to a composite ILE containing fish oil (38). An analysis assessing those using the composite ILE with fish oil for at least 12 months (17 out of 64 patients) reported an increase in the proportion of energy derived from ILEs (from 8 to 22%) and a corresponding reduction in energy supplied by glucose/dextrose (from 66 to 54%), maintaining stable alkaline phosphatase and triglyceride levels, and achieving improvements in other laboratory parameters [aspartate aminotransferase (AST), alanine aminotransferase (ALT), total bilirubin, and *α*-tocopherol] (38). It is also worth mentioning a further case in which hepatic fibrosis was reversed through rationalization of calories as well as administration of ILEs containing fish oil (45).

Generally, more data are available from the field of pediatric than adult PN concerning the use of composite ILEs containing fish oil to prevent or correct liver complications (46–49). Data stem from meta-analyses, clinical studies and clinical experience, and in general, they support the use of a composite ILE containing fish oil to prevent or reverse liver complications in pediatric patients requiring PN (46–49). Although the clinical data on ILEs containing fish oil for the prevention of IFALD in HPN patients is limited to case studies in adults or extrapolated from pediatric studies, the experts wanted to draw attention to these data, which suggest a clinically beneficial approach to one of the major threats associated with HPN, and formulated a corresponding statement (Table 1, statement 30). Nevertheless, management of other risk factors is also important, particularly infections (whether catheter-related infections or small intestinal bacterial overgrowth), in order to foster liver health during long-term PN (46–49).

The question of whether to use pure fish oil ILEs may arise within the context of IFALD. Pure fish-oil ILEs have been shown to be a valuable short-term rescue treatment in pediatric patients with cholestatic IFALD (46). Experience using pure fish oil as a potential therapeutic option for IFALD in adult patients is limited to a few, though promising, cases (50, 51). Additional research is needed to substantiate these largely anecdotal results, preferably testing the effectiveness of pure fish oil in comparison with standard care (50, 51).

## Composite ILEs in HPN and long-term PN use: clinical evidence

Overall, evidence remains relatively scarce concerning the effects of different ILEs on clinical or laboratory parameters for patients receiving HPN and/or during long-term PN (52). However, considerably more evidence is available from other situations in which PN is used, such as in surgical, hospitalized and critically ill adult patients (12, 53–56). In these patient populations, for instance, large meta-analyses and a network-meta-analysis have confirmed that ILEs containing fish oil have significant clinical advantages over ILEs without fish oil, including reduced risk of infection, and shorter intensive care unit (ICU) and hospital stays (12, 53–56). One of these meta-analyses also reported that ILEs containing fish oil had favorable effects on liver chemistry [AST, ALT, and *γ*-glutamyl transferase (GGT)], higher levels of the antioxidant *α*-tocopherol, lower levels for markers of inflammation such as tumor necrosis factor-alpha [TNF-*α*], and an improved fatty-acid profile (53).

A few small clinical studies and systematic reviews have investigated liver function and fatty-acid profiles comparing ILEs containing fish and/or olive oil with pure soybean-oil ILEs in patients receiving HPN (15, 52, 57). The newest and most complete systematic review to date included 295 patients from seven randomized controlled trials (RCTs), two prospective cohort studies and one cross-sectional study (52). All of the included studies compared different types of ILE, whereas the cross-sectional study also included patients prescribed lipid-free HPN. Across all studies, the main indications for HPN were short-bowel syndrome, Crohn’s disease, and vascular ischemia (52). In general, all ILEs were well tolerated, with no significant adverse effects. ILEs containing olive oil and/or fish oil were associated with a lower omega-6: omega-3 PUFA ratio, positive reductions in markers of liver function, and changes in blood and cell fatty-acid profiles. The longitudinal studies included in the meta-analysis (52) typically evaluated patients for 1–3 months, though one RCT published results after 12 months (58) and also long-term outcomes after a 5-year observation period (25). This last RCT included 88 patients, of whom 65 completed a 60-month visit, with patients randomized to receive either: (i) pure soybean oil; (ii) a blend of soybean oil and MCT at a ratio of 50:50; (iii) a blend of olive oil/soybean at a ratio of 80:20, or (iv) a blend of soybean oil/MCT/olive oil/fish oil at a ratio of 30:30:25:15. At the start of the study, variations in liver function parameters [AST, ALT, *γ*-glutamyl transpeptidase (GGTP), alkaline phosphatase, and median bilirubin] were observed across groups, but these measures normalized over the 12-month observation period (58). After 5 years of HPN, composite ILEs were found to be effective with a good safety profile, and by months 24 and 60 no significant differences were observed between groups for most liver function parameters (AST, ALP, GGTP, alkaline phosphatase) (25). However, in those receiving an ILE containing fish oil a decrease in median bilirubin concentration was observed at 60-months compared with baseline levels (25).

A recent RCT, the HPN with omega-3 (HOME) study, compared soybean oil/MCT at a ratio of 50:50 with soybean oil/MCT/fish oil at a ratio of 40:50:10 in HPN patients (59, 60). This study has raised awareness concerning challenges in patient recruitment associated with RCTs within HPN populations (59). A multicenter, multinational approach was chosen to try to meet the statistically required sample size (and also to increase external validity and the international generalizability of the results) (59, 60). For the assessment of the primary outcome (sum of changes in the liver function tests for bilirubin, AST, and ALT, from baseline to final visit), 160 patients (80 per study group) were needed according to statistical calculations. However, the study did not reach the planned sample sizes owing to insufficient patient recruitment and premature study termination, with only 74 patients being enrolled in total and so the primary analysis was underpowered (59). Over the observation period of 8–12 weeks, liver function parameters showed no clinically relevant differences between study groups, and remained within normal ranges. In the group of patients receiving ILE with fish oil, the omega-6: omega-3 PUFA ratio decreased and the median omega-3 index significantly increased to the desirable range (>8%) by the end of the study. This is relevant from a general health perspective as an omega-3 index greater than 8% has been associated with an approximately 30% reduced risk of fatal coronary heart disease when compared with an omega-3 index of less than 4% (59, 61). Taken together, there are promising clinical results in favor of using ILEs containing fish oil over pure soybean oil ILEs for HPN patients, but studies have shortcomings, and it would of course be warranted to have more solid evidence. Nevertheless, the experts at the summit considered it important to formulate a corresponding statement based on available evidence (Table 1, statement 26), to weigh clinical requirements and the hurdles to obtaining definitive data for this vulnerable patient group (Table 1, statement 39).

## HPN and palliative nutrition

Patients with end-stage cancer may also be given HPN, though the number of cancer patients receiving HPN differs considerably between countries (2). In general, nutrition plays a crucial role in cancer care given that malnutrition is a common feature among cancer patients. Both the presence of the tumor and medical and surgical anticancer treatments may promote malnutrition, with an estimated 10–20% of cancer patients dying from the consequences of malnutrition rather than from their tumor (62). ESPEN recommends home enteral nutrition (EN) or HPN in patients with chronic insufficient dietary intake and/or uncontrollable malabsorption (62). Some patients may use PN as their sole source of nutrition, others may receive a combination of PN and oral nutrition/EN (Table 1, statement 32) (9). In cancer care, nutrition support is recommended during hospital admission and discharge (62).

ILEs are an integral part of PN (Table 1, statement 25) (12). In those with cancer, it may be particularly advantageous to partially replace more glucose/dextrose with lipids in PN than in other types of patients, owing to disease-induced alteration of carbohydrate metabolism and an increased need for lipids within this population (62). The increased need for lipid in oncology patients is the result of cancer induced modifications to lipid metabolism (63). Early investigations, for example, described an efficient mobilization and oxidation of endogenous fat as a fuel source in cancer patients (64–66). The metabolic clearance of ILEs increases in both weight-stable and (even more so) in weight-losing cancer patients compared with healthy volunteers (62, 66, 67).

As outlined earlier, composite ILE alternatives to pure soybean-oil ILEs result in less inflammation and immune suppression, and have greater antioxidant effects, and so are nowadays often the preferred option within clinical practice (12). ILEs containing fish oil have distinct biological effects, as explained earlier, and are associated with clinical advantages over ILEs without fish oil, including reduced risk of infection, and shorter ICU and hospital stays (12, 53–56). Two analyses showing these clinical benefits included gastrointestinal cancer surgery patients, among others (53, 56). A meta-analysis of cancer surgery patients reported promising results in favor of ILEs with fish oil compared with ILEs without fish oil, including a lower rate of infectious complications, shorter hospital stays, and favorable effects on immune markers (CD4 + and CD8 + cells) (68). Other meta-analyses have investigated nutrition containing omega-3 PUFAs in cancer patients without distinguishing between the route of administration (EN and/or PN) (69–71). Overall, the results of these analyses are difficult to interpret, given the fundamental differences in duration and dosing of omega-3 PUFAs in the mixture of PN and EN studies (69–71). Subgroup analyses looking at patients receiving omega-3 PUFA as part of their PN reported favorable effects on the inflammatory marker TNF-*α* (70, 71), as well as reducing the rate of infectious and non-infectious complications (70), but could not find significant effects on other (inflammatory) markers such as interleukin-6 (IL-6) or C-reactive protein (CRP) (71). In summary, there are initial promising results in favor of the use of ILEs containing fish oil in patients with cancer requiring PN, but further evidence is needed to draw firm conclusions.

## Future perspectives

In this section we consider the following main questions, that are particularly relevant to the use of ILEs as part of long-term/home parenteral nutrition: (i) why is the generation of definitive clinical studies/RCTs particularly difficult within this field? (ii) what alternative approaches to traditional RCTs might be useful? and (iii) which approaches to take as part of current clinical practice in the absence of definitive data?

RCTs are generally considered the gold-standard method for generating clinical data (72). In the aforementioned HOME study in CIF patients, a sensible approach was taken to facilitate recruitment into this RCT comparing different ILEs. However, this multicenter protocol turned out to be challenging for some centers, as was the requested minimum ILE target of 3 g/kg/week, and beyond that the study fell into the period of the COVID-pandemic which also impeded recruitment (59). More importantly, CIF is a rare disease with estimated prevalence rates of 5 to 80 cases per million people (1, 4, 5). At the time of diagnosis, patients are also dealing with a complex set of medical issues that led to the development of CIF. Additionally, they are also coping with the psycho-social aspects of CIF, the requirement for HPN, as well as significant modification of their dietary intake. These aspects make it very difficult to carry out RCTs with sufficient power to answer key questions within the field of HPN.

In other areas where PN is used, such as in critical illness, there is increased appetite to perhaps rethink the approach to data generation as studies evaluating the relationship between health outcomes and lipids used in PN are often inconclusive (73). RCTs typically include low patient numbers or have other methodological shortcomings hampering the generation of robust data, other factors such as heterogeneity of patient populations, or a suboptimal understanding of biological mechanisms, which may all contribute to inconclusive evidence (73). Optimized trial designs and/or greater use of biomarkers have been suggested to overcome these challenges (73). It is debatable whether greater use of biomarkers would be a promising approach for HPN, not least because biomarkers already play an important role in HPN studies. It certainly may be worth rethinking trial concepts for HPN studies. For example, adaptive platform trials might be worth considering as potential alternative to replace traditional rigid and costly RCTs (74). Other possible approaches include those based on natural history or other real-world registers, as used for rare diseases (75), along with the formation of registry networks to give the option of data pooling (76). To our knowledge, none of these concepts have been explored so far to a large extent in clinical nutrition, so we do not know what potential they may have for patients receiving HPN.

The “Home Artificial Nutrition & Chronic Intestinal Failure” (HAN-CIF) initiative by ESPEN may offer the prospect to generate real-word data within the HPN field. The registry includes more than 16,000 patients worldwide, and so far has served primarily to characterize and classify patients with CIF (77). In summary, conclusive data within the field of clinical nutrition remain a long way off, and so in the meantime the existing body of evidence must provide the basis for clinical care. Where evidence is lacking, expert recommendations have to suffice. For ILEs in HPN, respective translation into clinical care has been done by formal guideline committees (2, 6, 13), and in a more condensed manner by the Lipid Summit experts (12). The consensus statements from the Lipid Summit aim to summarize aspects mostly relevant for everyday clinical care; those relevant to the HPN setting are listed in Table 1 (12).
